# Types 1 diabetes mellitus and Graves’ disease in Sanjad-Sakati syndrome: a case report in Saudi Arabia

**DOI:** 10.3389/fendo.2026.1920847

**Published:** 2026-09-30

**Authors:** Ghada Almasri, Alaa Ahmed Almubayedh, Maryam Zaitoun, Nawal Almutairi, Abdulhameed Alsaheel

**Affiliations:** 1 College of Medicine, Alfaisal University, Riyadh, Saudi Arabia; 2 Pediatric Endocrinology Department, Obesity, Endocrine and Metabolism Center, King Fahad Medical City, Riyadh, Saudi Arabia

**Keywords:** diabetes mellitus, hyperthyroidism, hypoparathyroidism-retardation-dysmorphism syndrome (HRDS), hypothyroidism, Sanjad-Sakati syndrome (SSS), Saudi Arabia

## Abstract

Sanjad-Sakati syndrome (SSS) is a rare autosomal recessive disorder caused by TBCE gene variants and characterized by congenital hypoparathyroidism, severe growth retardation, developmental delay, and dysmorphic features. We report a nine-year-old Saudi boy with genetically confirmed SSS due to a homozygous 12-bp deletion in exon 3 of the TBCE gene (c.155_166del12bp; p.S52_G55del), who presented with hypocalcemic convulsions at three weeks of age. At three years, he developed type 1 diabetes mellitus (T1DM) with profound insulin deficiency and positive anti-islet cell and anti-GAD-65 antibodies. At four years, he developed Graves’ disease, confirmed by hyperthyroidism, positive TSH receptor antibodies, and increased thyroid vascularity, followed by hypothyroidism requiring levothyroxine. Severe bilateral medullary nephrocalcinosis was also identified. This case highlights the unusual coexistence of TBCE-related SSS, autoimmune T1DM, and Graves’ disease, raising the possibility of potential immune dysregulation, although causality cannot be established. Longitudinal endocrine surveillance, particularly of thyroid function, may be warranted in patients with SSS.

## 1 Introduction

Sanjad-Sakati Syndrome (SSS), also known as hypoparathyroidism-retardation-dysmorphism (HRD), is a rare autosomal recessive disease (*OMIM # 241,410*) first reported by Sanjad et al. in Saudi Arabia in 1988 (1, 2). It is a very uncommon disorder, predominantly found in children of Arab origin, although some cases of non-Arabic descent have also been documented. The main endocrine manifestation in SSS is hypoparathyroidism (3). Affected children may also present with signs and symptoms such as significant delay in developmental milestones, including both gross and fine motor skills (4). Additionally, they may present with retardation in physical and mental growth, seizures, as well as craniofacial and orodental deformities (4). Until now, the most common cause of mortality in SSS is infection as a result of hyposplenism (4).

SSS occurs due to a mutation in the tubulin cofactor E (*TBCE*) gene located in chromosome 1q42-q43 (5). In Middle Eastern populations, SSS most commonly results from homozygous pathogenic variants in TBCE, often reflecting consanguinity (6). This gene codes for a molecular chaperone which normally joins two different subunits, alpha-tubulin and beta-tubulin, in a process known as heterodimerization. As a result, a quaternary structure is produced which is essential for the assembly pathways of microtubules and tubulin cytoskeleton proteins responsible for cellular trafficking, signal transduction, and cell migration (6, 7). Pathogenic TBCE variants impair tubulin folding and microtubule assembly, disrupting cellular processes that may be particularly important for parathyroid development. Consequently, cytoskeletal fibers such as microtubules will be affected hence affecting the cell function (6). TBCE and proper microtubule formation could be key to the development of the parathyroid gland, which may explain the consistent association between TBCE mutations and hypoparathyroidism in this syndrome (8).

Although hypoparathyroidism is the hallmark endocrine manifestation of SSS, other endocrine abnormalities have been increasingly recognized. In a retrospective study done in 2021, David et al. investigated the endocrine profile of 58 patients known to have SSS by measuring thyroid function, insulin-like growth factor (IGF)-1, and gonadotropin concentrations (9). In 21 of the 58 patients, serum IGF-1 was low for age or undetectable in all of them (9). Hypoglycemic episodes requiring hospitalization (<50 mg/dL) occurred in 55% of the 58 patients (9). Serum thyrotropin (TSH) and free thyroxine (FT4) were measured in 44 patients, with 36% diagnosed with hypothyroidism (9). In 2016, another retrospective study in Riyadh assessed the thyroid status of 18 patients with SSS, finding that about a third of these patients had hypothyroidism due to autoimmune thyroiditis (10).

Reports of diabetes mellitus in SSS are limited. A case from Kuwait described the development of T1DM in a child with SSS in temporal association with COVID-19 infection (11). More recently, an international rare disease registry reported nonautoimmune insulin-requiring diabetes in 2 of 127 patients with SSS. The same registry reported thyroid disorders in 22 patients, with hypothyroidism accounting for 21 cases and Graves’ disease for one case (12). These findings suggest that endocrine abnormalities may be more diverse than previously recognized, although the relationship between SSS and autoimmune disease remains uncertain.

Although cases of autoimmune thyroiditis and hypoglycemia have been reported in SSS, the coexistence of antibody-positive T1DM and Graves’ disease has not been previously reported in a patient with SSS (9, 10). We present a case of a nine-year-old male with SSS who was admitted to King Fahad Medical City, Riyadh, with T1DM and Graves’ disease.

## 2 Case presentation

A nine-year-old Saudi boy with genetically confirmed SSS was referred to King Fahad Medical City in Riyadh, Saudi Arabia. He had initially presented with hypocalcemic convulsions at three weeks of age. He was diagnosed with SSS at birth, characterized by small-body size, short stature, deeply sunken eyes, micrognathia, depressed nasal bridge, and large, floppy ears. The patient was born to consanguineous Saudi parents via full-term normal vaginal delivery. Genetic testing identified a homozygous deletion mutation in exon 3 of the *TBCE* gene (c.155_166del12bp; p.S52_G55del), confirming the molecular diagnosis of SSS.

At initial presentation, biochemical testing confirmed hypoparathyroidism, with low parathyroid hormone (PTH) levels, hypocalcemia (Ca = 1.3 mmol/L, reference range: 2.2-2.7 mmol/L), and elevated phosphorus levels (PO4 = 3.5 mmol/L, reference range: 1.54-2.72 mmol/L) (**Table 1**). He was treated with oral calcium, alfacalcidol, and anticonvulsive drugs for symptomatic management. Since birth, he has experienced hypocalcemic convulsions and frequent hospitalizations for chest infections and pneumonia. There was also a noted delay in speech and walking. At nine years of age, the patient remained severely growth restricted, with a weight of 6.4 kg (Z-score −22.1) and height of 73 cm (Z-score −10), both below the third percentile.

**Table 1:** Biochemical parameters at initial presentation.

Parameter	Result	Reference range
Serum calcium (mmol/L)	1.3	2.2 - 2.7
Serum phosphate (mmol/L)	3.5	1.54 - 2.72

### 2.1 Type 1 diabetes mellitus

At three years, his parents noticed a decline in their child’s activity level. They denied any signs of polyuria, polydipsia, weight loss, abdominal pain, nausea, or headache. However, there was a positive family history of T1DM in his older brother.

At presentation, random blood sugar level was high at 30.8 mmol/L, and his initial glycated hemoglobin (HbA1c) was 11.5%. Laboratory tests revealed severe insulin deficiency with C-peptide levels <0.003 nmol/L (reference range: 0.37-1.47 nmol/L) and serum insulin <1.39 pmol/L (reference range: 17.8–173 pmol/L) (**Table 2**). The patient’s tests for anti-islet cell antibodies and anti-glutamic acid decarboxylase 65 antibodies (anti-GAD-65) were positive. Electrolytes and thyroid function were normal.

**Table 2:** Biochemical parameters at three years of age.

Parameter	Result	Reference range
Random blood sugar (mmol/L)	30.8	<6.9
C-peptide (nmol/L)	<0.003	0.37 - 1.47
Serum insulin (pmol/L)	<1.39	17.8 - 173

The patient was diagnosed with T1DM without diabetic ketoacidosis (DKA) and started on insulin. At present, he adheres to a multiple daily injection regimen with insulin degludec and insulin aspart, totaling (0.8-1.0 units/kg/day). During follow-up, his HbA1c remained between 9 and 10%, with no microvascular complications, significant hypoglycemia or DKA since the initial diagnosis of diabetes.

### 2.2 Graves’ disease and subsequent hypothyroidism

At four years of age, the patient developed hyperthyroidism, manifested as progressive painless neck enlargement and a 1.1-kg weight loss over one month, from 6.67 kg to 5.5 kg. His mother noted no issues with swallowing or breathing. There was no reported history of steatorrhea, abdominal pain, palpitations, muscle pain, sleep disturbance, or changes in diet or physical activity.

Physical examination demonstrated fatigue and sinus tachycardia without proptosis or lymphadenopathy. Laboratory investigations revealed a normal renal profile, while the complete blood count showed leukocytosis of 16 × 10^9^/L (reference range: 4.3-11.3 × 10^9^/L), an absolute neutrophil count of 9.28 × 10^9^/L (reference range: 1.35-7.5 × 10^9^/L), a platelet count of 333 × 10^9^/L (reference range: 155-435 × 10^9^/L) and a hemoglobin level of 13.9 g/dL (reference range: 11–15 g/dL).

Thyroid-function testing showed a suppressed thyroid-stimulating hormone (TSH): <0.01 mIU/L (reference range: 0.5-5.1 mIU/L), elevated free thyroxine (free T4): 28.5 pmol/L (reference range: 9–19 pmol/L), and positive TSH receptor antibody (TRAb). Tests also showed increased levels of anti-thyroglobulin antibody: 16.3 (reference range: 0-4.11 IU/ml) and anti-thyroid peroxidase antibody: >1000 IU/ml (reference range: 0-5.61 IU/ml). Ultrasonography showed a mildly enlarged, heterogeneous thyroid with increased vascularity on color Doppler (**Figure 1**).

**Figure 1:**
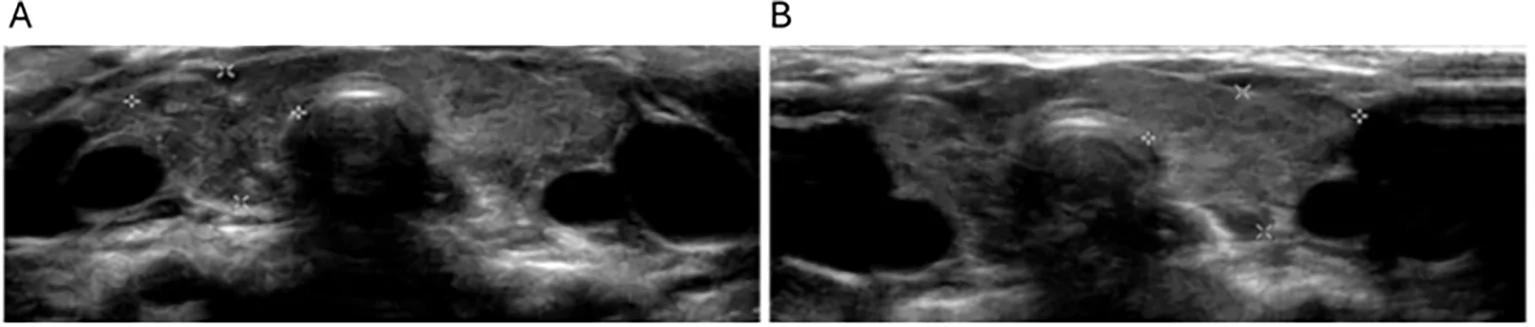
Thyroid ultrasonography demonstrating mild enlargement and heterogeneous echotexture of the thyroid gland, involving the right lobe **(A)** and left lobe **(B)**, consistent with the clinical diagnosis of Graves’ disease.

Based on these findings, the patient received atenolol 5 mg/day and carbimazole 1.25 mg/day. Atenolol was discontinued one week later. Hyperthyroidism remitted after two years, but he later developed hypothyroidism (TSH 15 mIU/L, free T4 6.25 ng/dL) (**Table 3**) and was started on levothyroxine (25 mcg daily). His thyroid function has remained stable with continued therapy.

**Table 3:** Laboratory parameters at four years of age.

Parameter	Result	Reference range
White blood cell count (x10^9^/L)	16	4.3 - 11.3
Absolute neutrophil count (10^9^/L)	9.28	1.35 - 7.5
Platelet count (10^9^/L)	333	155 - 435
Hemoglobin (g/dL)	13.9	11 - 15
Thyroid-stimulating hormone (mIU/ml)	<0.01	0.5 - 5.1
Free thyroxine (pmol/L)	28.5	9 - 19
Anti-thyroglobulin antibody (IU/ml)	16.3	0 - 4.11
Anti-thyroid peroxidase antibody (IU/ml)	>1000	0 - 5.61

Given the coexistence of T1DM and autoimmune thyroid disease, additional autoimmune investigations were performed. Celiac disease-associated antibodies and adrenal antibodies were negative. No additional comprehensive immunological or organ-specific autoimmune profiling was performed. Therefore, the available investigations do not exclude the possibility of other autoimmune conditions.

There was no documented history of symptomatic or diagnosed COVID-19 infection before the onset of T1DM. However, SARS-CoV-2 infection was not specifically excluded using virological or serological testing; therefore, a previous asymptomatic or unrecognized infection cannot be definitively ruled out.

### 2.3 Nephrocalcinosis

During follow-up, renal ultrasound revealed severe bilateral diffuse medullary nephrocalcinosis, despite normal renal function (**Figure 2**). Hydrochlorothiazide was initially prescribed, but the treatment was later adjusted to include amlodipine due to hypertension.

**Figure 2:**
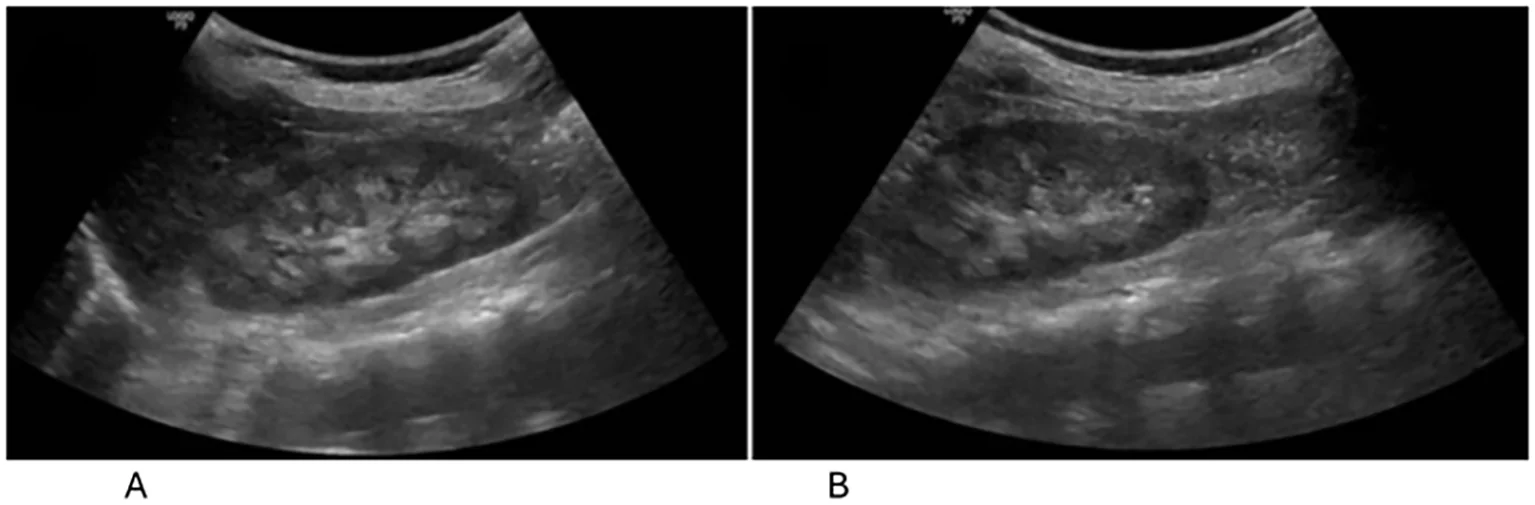
Renal ultrasonography demonstrating severe bilateral diffuse medullary nephrocalcinosis involving the right kidney **(A)** and left kidney **(B)**.

The patient’s clinical course is summarized in **Table 4**.

**Table 4:** Clinical timeline of our patient with SSS.

Age	Clinical event	Key findings/intervention
3 weeks	Presentation with hypocalcemic convulsions	Hypocalcemia, hyperphosphatemia, low PTH; SSS diagnosed; calcium and alfacalcidol started
3 years	Type 1 diabetes mellitus diagnosed	Glucose 30.8 mmol/L, HbA1c 11.5%, markedly low C-peptide, positive anti-islet cell and anti-GAD-65 antibodies; insulin initiated; no DKA
4 years	Graves’ disease diagnosed	Goiter, weight loss, suppressed TSH, elevated FT4, positive TRAb; carbimazole initiated
4–6 years	Graves’ disease treatment	Carbimazole continued; hyperthyroidism subsequently remitted
After remission	Hypothyroidism developed	Elevated TSH and low FT4; levothyroxine initiated
During follow-up	Nephrocalcinosis identified	Severe bilateral medullary nephrocalcinosis despite preserved renal function
9 years	Current status	Stable thyroid function on levothyroxine; ongoing insulin therapy; persistent severe growth restriction

## 3 Discussion

The principal finding in this case is the coexistence of genetically confirmed TBCE-related SSS with two autoimmune endocrine disorders, T1DM and Graves’ disease. Although endocrine abnormalities beyond congenital hypoparathyroidism have increasingly been recognized in SSS, the relationship between SSS and autoimmune endocrinopathy remains poorly understood. The longitudinal development of autoimmune T1DM followed by Graves’ disease in our patient provides an unusual clinical phenotype and raises the possibility of broader endocrine or immune dysregulation.

In a study of 58 patients, about half exhibited at least one endocrinopathy, and about 14% presented with two or more. These manifestations often appear in adolescence, supporting the idea that endocrine dysfunction in SSS may progress over time (9).

### 3.1 SSS and type 1 diabetes mellitus

Diabetes mellitus has been reported only rarely in SSS. A 2022 report from Kuwait described T1DM developing in a child with SSS following COVID-19 infection (11). Several studies have shown that SARS-CoV-2 can directly damage pancreatic tissue, worsening hyperglycemia and even inducing diabetes in previously non-diabetic individuals (13). In our patient, T1DM developed in the absence of a documented preceding COVID-19 infection. However, because neither SARS-CoV-2 PCR nor serological testing was performed specifically to exclude previous infection, an asymptomatic or unrecognized infection cannot be definitively ruled out.

The diagnosis of T1DM in our patient was supported by several findings: marked hyperglycemia, an HbA1c of 11.5%, profound insulin deficiency with markedly reduced C-peptide and insulin concentrations, and positive anti-islet cell and anti-GAD-65 antibodies. These findings distinguish the patient’s diabetes from nonautoimmune diabetes.

This distinction is relevant because the recent international SSS registry reported insulin-requiring diabetes in two patients, but these cases were classified as nonautoimmune diabetes (12). Thus, our patient represents a different phenotype characterized by serologically supported autoimmune β-cell destruction.

Another noteworthy feature was the absence of DKA despite severe hyperglycemia, markedly elevated HbA1c, and profound insulin deficiency. Although DKA is a common presentation of childhood T1DM, not all children present with ketoacidosis. the risk may be influenced by the timing of diagnosis, duration of hyperglycemia, and individual disease progression (14). Given the limited number of reported cases of diabetes in SSS, no conclusions can currently be drawn regarding whether DKA risk differs from that in the general pediatric population.

The positive family history of T1DM in the patient’s older brother is also relevant. It provides an alternative explanation for the development of autoimmune T1DM independent of SSS and further emphasizes why a causal relationship between the TBCE variant and autoimmunity cannot be inferred from this case.

Other studies showed that 55% (32/58) of patients experienced hypoglycemic events, particularly during concurrent illnesses, with insulin suppression and elevated cortisol observed in most cases (9).

### 3.2 SSS and thyroid disease

The identification of Graves’ disease in our patient is noteworthy given its rarity in SSS. Most reports describe autoimmune hypothyroidism as the more common thyroid abnormality, with one study evaluating 18 patients with SSS in Riyadh and found abnormal thyroid-function tests in six patients, including five with subclinical hypothyroidism and one with overt hypothyroidism. Thyroid autoantibodies were positive in four patients, leading the authors to attribute most of the thyroid abnormalities to autoimmune thyroiditis and to recommend regular thyroid-function and thyroid-autoantibody screening (10).

Similarly, a retrospective study of 58 patients reported hypothyroidism in 36% of patients who underwent thyroid-function testing (9). More recent registry data further support the occurrence of thyroid disease in SSS. In an international rare disease registry, thyroid disorders were identified in 22 of 127 patients (17.3%), of whom 21 had hypothyroidism and only one had Graves’ disease (12). This variation raises questions about the diversity of thyroid disorders within the SSS population.

The present case is particularly notable because the patient developed Graves’ disease rather than the autoimmune hypothyroidism more commonly described in previous SSS cohorts. Graves’ disease and Hashimoto thyroiditis represent different manifestations of thyroid autoimmunity. Graves’ disease is primarily mediated by stimulatory autoantibodies against the TSH receptor (15), whereas autoimmune hypothyroidism is associated predominantly with destructive thyroid autoimmunity and antibodies against thyroid antigens such as TPO and thyroglobulin (16).

In our patient, the diagnosis of Graves’ disease was supported by suppressed TSH, elevated free T4, positive TRAb, goiter, and increased thyroid vascularity on Doppler ultrasonography. The subsequent development of hypothyroidism may represent the evolution of autoimmune thyroid disease, the effect of antithyroid treatment, or a combination of both. The occurrence of Graves’ disease therefore suggests that thyroid autoimmunity in SSS may not be restricted to the predominantly hypothyroid phenotype described in earlier studies.

### 3.3 Potential relationship between TBCE dysfunction and autoimmunity

There is also evidence that some individuals with TBCE-related disease may have immune abnormalities, including reports describing combined immune deficiency in patients with hypoparathyroidism-retardation-dysmorphism syndrome (17). However, there is currently no established evidence demonstrating that TBCE mutations directly cause autoimmune disease or specifically predispose patients to T1DM or Graves’ disease.

One hypothesis is that disruption of microtubule-dependent cellular processes could potentially influence immune-cell development, trafficking, activation, or immune tolerance. Nevertheless, this remains speculative, and the presence of autoimmune endocrinopathies in our patient may also represent coincidental coexistence of common autoimmune diseases in childhood. A causal relationship between the TBCE mutation and autoimmunity cannot be inferred from this single case. Functional immune profiling, cytokine studies, autoantibody profiling, and mechanistic cellular studies were not performed in our patient.

### 3.4 Relationship to autoimmune polyendocrine syndromes

The coexistence of T1DM and Graves’ disease also raises consideration of an autoimmune polyendocrine syndrome (APS) phenotype. T1DM and autoimmune thyroid disease, including Graves’ disease, are recognized components of autoimmune polyendocrine syndromes, particularly APS type 2 and APS type 3 variants (18). However, our patient does not fit the classical definition of APS type 1 because the underlying congenital hypoparathyroidism is attributable to a pathogenic TBCE variant rather than an established autoimmune mechanism.

Similarly, the absence of adrenal autoimmunity and adrenal insufficiency argues against APS type 2. Therefore, the patient’s phenotype is better considered SSS with coexisting autoimmune T1DM and Graves’ disease rather than a classical APS. Nevertheless, the clinical overlap raises the possibility that some patients with SSS may develop an APS-like clustering of autoimmune endocrine disorders.

### 3.5 Nephrocalcinosis

Hypocalcemia is the most common complication in SSS, often leading to iatrogenic hypercalcemia. Both hypercalcemia and vitamin D treatment increase the risk of nephrocalcinosis (19). One study found nephrocalcinosis and nephrolithiasis in 47% of SSS patients within their first year, with most cases diagnosed in the second decade of life (5). Another study found renal calcification in 66% of 24 patients with SSS (20).

Severe bilateral diffuse medullary nephrocalcinosis developed in our patient despite preserved renal function, emphasizing the importance of monitoring urinary calcium excretion and renal imaging during long-term management of hypoparathyroidism.

### 3.6 Strengths and limitations

This report has several strengths. First, the patient has a molecularly confirmed diagnosis of SSS with a pathogenic TBCE deletion, providing a robust genetic basis for the underlying syndrome. Second, the longitudinal follow-up documents the sequential development of several endocrine abnormalities over nine years. Third, the diagnosis of autoimmune T1DM was supported by both profound insulin deficiency and positive pancreatic autoantibodies. Similarly, Graves’ disease was supported by biochemical hyperthyroidism, positive TRAb, goiter, and increased thyroid vascularity on ultrasonography. The detailed clinical and biochemical characterization therefore provides stronger evidence for the autoimmune diagnoses than would be possible from clinical features alone.

Several limitations should also be acknowledged. The rarity of SSS and the occurrence of multiple endocrine disorders in a single patient prevent determination of whether this phenotype occurs more frequently than expected in the general population. Moreover, no mechanistic investigations were performed to explore a potential relationship between the TBCE variant and immune dysregulation. In particular, detailed immunophenotyping, cytokine profiling, functional immune assays, and cellular studies were not performed. The autoimmune evaluation was also limited to the investigations available in the clinical record, including pancreatic autoantibodies, thyroid autoantibodies, adrenal antibodies, and celiac serology; therefore, other autoimmune conditions cannot be completely excluded. Additionally, the absence of documented COVID-19 infection was based on clinical history rather than systematic virological or serological testing, and previous asymptomatic infection cannot therefore be definitively excluded. The patient’s family history of T1DM provides an alternative potential explanation for autoimmune diabetes independent of SSS. Finally, as with all single-case reports, the coexistence of T1DM and Graves’ disease should be interpreted as an observation that generates hypotheses rather than evidence of causality.

## 4 Conclusion

This case describes the unusual coexistence of genetically confirmed TBCE-related Sanjad–Sakati syndrome, antibody-positive type 1 diabetes mellitus, and Graves’ disease in a child. Although thyroid disease, particularly hypothyroidism, has been reported in SSS, Graves’ disease and autoimmune T1DM appear to be uncommon in this condition. The coexistence of these disorders raises the possibility of an expanded endocrine phenotype, but a causal relationship between the TBCE variant and autoimmunity cannot be established from a single case, particularly in the presence of a family history of T1DM. Longitudinal monitoring of thyroid function is reasonable in patients with SSS, whereas screening for diabetes and other autoimmune disorders should be guided by symptoms, family history, and clinical findings. Further multicenter studies are needed to determine whether autoimmune endocrinopathies occur more frequently in patients with SSS.

## Data Availability

The original contributions presented in the study are included in the article/supplementary material. Further inquiries can be directed to the corresponding author.
